# Clomiphene citrate versus high doses of gonadotropins for *in vitro* fertilisation in women with compromised ovarian reserve: a randomised controlled non-inferiority trial

**DOI:** 10.1186/1477-7827-10-114

**Published:** 2012-12-18

**Authors:** Guido Ragni, Paolo E Levi-Setti, Rubens Fadini, Claudio Brigante, Claudia Scarduelli, Federica Alagna, Veronica Arfuso, Mario Mignini-Renzini, Massimo Candiani, Alessio Paffoni, Edgardo Somigliana

**Affiliations:** 1 Infertility Unit, Fondazione IRCCS Cà Granda, Ospedale Maggiore Policlinico, Milan, Italy; 2 Department of Gynecology and Reproductive Medicine, IRCCS Istituto Clinico Humanitas, Rozzano, Italy; 3 Reproductive Medicine Unit BIOGENESI, Istituti Clinici Zucchi, Monza, Italy; 4 Obstetrics and Gynecology Unit, Università Vita-Salute, Fondazione Ospedale San Raffaele, Milan, Italy

**Keywords:** Poor responder, Ovarian reserve, Clomiphene citrate, IVF

## Abstract

**Background:**

The aim of the present randomised controlled non-inferiority trial is to test whether in women with compromised ovarian reserve requiring *in vitro* fertilisation, a protocol of ovarian stimulation using exclusively clomiphene citrate performs similarly to a regimen with high doses of gonadotropins.

**Methods:**

Women with day 3 serum FSH > 12 IU/ml on at least two occasions or previous poor response to hyper-stimulation were recruited at four Italian infertility units. Selected women were allocated to clomiphene citrate 150 mg/day from day 3 to day 7 of the cycle (n=145) or to a short protocol with GnRH agonist 0.1 mg and recombinant FSH 450 IU daily (n=146). They were randomised by means of a computer-generated list into two groups. The study was not blinded. The main outcome of the study was the delivery rate per started cycle.

**Results:**

The study was interrupted after the scheduled two years of recruitment before reaching the sample size. 148 women were allocated to clomiphene citrate and 156 to the short protocol with high doses of gonadotropins; 124 and 125 participants were analysed in the groups, respectively. Women allocated to high doses of gonadotropins retrieved more oocytes and had a higher probability to perform embryo-transfer. However, the chances of success were similar. The delivery rate per started cycle in women receiving clomiphene citrate and high-dose gonadotropins was 3% (n=5) and 5% (n=7), respectively (p=0.77). The mean estimated cost per delivery in the two groups was 81,294 and 113,107 Euros, respectively. No side-effects or adverse events were observed.

**Conclusions:**

In women with compromised ovarian reserve selected for *in vitro* fertilisation, ovarian stimulation with clomiphene citrate or high-dose gonadotropins led to similar chances of pregnancy but the former is less expensive.

**Trial registration:**

Trial registered on http://www.clinicaltrials.gov (NCT01389713)

## Background

Management of women with compromised ovarian reserve selected for *in vitro* fertilisation (IVF) is challenging. Affected women retrieve less oocytes, have less embryos for transfer and their chances of success are generally markedly lower. Not infrequently, their cycles have to be cancelled due to the absence of follicular growth, the lack of oocytes retrieved or because of failure to develop embryos [1-4]. Several strategies have been proposed to overcome this frustrating situation. They include an increase in the starting doses of gonadotropins, the use of regimens that are alternative to the long protocol such as the short protocol or the GnRH antagonist protocol or the natural cycle and the addition of LH or HCG, Growth Hormone (GH) or Growth Hormone Releasing Factor (GHRF), pyridostigmine, L-arginine, androgens, clomiphene citrate or letrozole [1-5]. Evidence for these approaches is however scarce and results have been disappointing. In light of this uncertainty, most physicians simply adopt a traditional and intuitive attitude consisting of increasing the starting dose of gonadotropins (typically 300–450 IU per day) [1]. However, this choice contrasts with the currently growing importance given to the idea of friendly IVF. There is increased attention in securing IVF with a low intervention level, low risks and few side effects [6]. Moreover, women with compromised ovarian reserve selected for IVF should not be exclusively viewed as a therapeutic challenge. The low rate of success and the increased costs of alternative protocols (in particular those entailing the use of high doses of gonadotropins) also pose an economical dilemma. One has to wonder about the rationale of employing expensive regimens that are not supported by scientific evidence.

On these bases, we designed a prospective multicentre non-inferiority Randomised Controlled Trial (RCT) comparing clomiphene citrate and a short protocol with high doses of gonadotropins in women with compromised ovarian reserve requiring IVF. The main aim was to test the hypothesis that a low-cost regimen of exclusively clomiphene citrate is similarly effective or only slightly worse than a regimen with high doses of gonadotropins.

## Methods

Four Italian centres participated in this study. They were the infertility units of the following institutions: 1) Fondazione Cà Granda, Ospedale Maggiore Policlinico, Milan; 2) Istituto Clinico Humanitas, Rozzano; 3) Istituti Clinici Zucchi, Monza; 4) Università Vita-Salute, Fondazione Ospedale San Raffaele, Milan. Patients referring to these units between September 2008 and August 2010 and selected for IVF were evaluated for study entry. Inclusion criteria were similar to those used in previous trials that established efficacy of the standard high dose treatment [1] and were as follows: 1) indication to IVF-ICSI; 2) age 18–42 years; 3) day 3 serum FSH > 12 IU/ml on at least two occasions or previous poor response (≤ 3 oocytes with a conventional stimulation protocol) in a previous IVF cycle. Exclusion criteria were as follows: 1) number of previous IVF cycles ≥ 3; and 2) cycles requiring the use of spermatozoa from MESA-TESE procedures. Before entering the study, the protocol was explained to patients and a written consent was obtained from patients agreeing to participate. Consent forms and the protocol were approved by the local ethical committees. The study was registered on http://www.clinicaltrials.gov with ref. NCT01389713.

Women were evaluated for study entry the month before the IVF cycle. If eligible and consenting to participate, they were randomised by means of a computer-generated list into two groups. An independent list was established for each study centre. Sealed opaque envelopes containing treatment allocation were opened after inclusion. Patients and physicians were not blinded to the treatment allocation. The two arms of the study were as follows: 1)clomiphene citrate oral tablets (Clomid®; Bruno Farmaceutici, Rome, Italy)at the dose of 150 mg/day from day 3 to day 7 of the cycle; or 2) daily s.c. injections of triptoreline (Decapeptyl®; 0.1 mg, Ipsen, Milan, Italy) started on day one or two of the menstrual cycle and 450 IU of s.c. recombinant FSH (Gonal F®; 450–900 UI pen; Merck Serono, Bari, Italy) from day 3 of the cycle. The standard treatment was administered according to that established in previous trials [1,3]. Subjects of both groups were monitored similarly. Transvaginal US scans were performed on days 3 and 7 of the cycle in all women. Subsequent scans were individualized based on ovarian response. Serum levels of peripheral oestrogens and progesterone were done if deemed necessary. The hCG triggering of ovulation (Ovitrelle®; 250 μg, sc; Merck Serono, Bari, Italy) was performed when ultrasound revealed at least one follicle with a mean diameter ≥ 18 mm. Subjects underwent ovum pick-up 36 hours later. The dose of recombinant FSH could not be increased but could be reduced in cases of hyper-response. Cycles were cancelled due to hyper-response (>20 follicles with a mean diameter >10 mm at the time of hCG) or low response (absence of follicular growth) or inadequate follicular development (arrest of follicular growth or premature luteinisation). Standard IVF or Intracytoplasmic Sperm Injection (ICSI) procedures were performed according to standardised criteria [7] and embryo transfer was executed on day 2 or 3. Luteal phase supplementation consisted of vaginal micronised progesterone tablets (Progeffik® 200 mg, Effikitalia, Milan, Italy) 200 mg twice a day for 12 days starting the day of oocytes retrieval. Medications were free of charges for the patients of both groups since they are routinely supported by the public health system in our region. Women were enrolled for one treatment cycle. Clinical pregnancy was defined as the ultrasound visualisation of at least one intrauterine gestational sac. Delivery refers to viable infants born after 24-weeks gestation. First pregnancy ultrasound was performed in the participating infertility units whereas the course of pregnancy was assessed through telephone contact and by consulting charts of the obstetrical departments of the participating institutes.

The main outcome of the study was the delivery rate per started cycle. The sample size was calculated based on the following assumptions: 1) study design was a non-inferiority trial; 2) type I error: one-sided 95% confidence interval (CI); 3) study power = 80%; and 4) expected delivery rate in the standard treatment group (high doses of gonadotropins) = 5%. This information was based on a previous study of our group in this specific population [5]; 5) largest difference to be regarded as negligible in favour of the standard treatment = 5%. On this basis, we calculated a sample size of 472 subjects (236 per arm). Based on an estimate made before initiating the study on the frequency of the studied condition in the participating centres, a recruitment period of two years was deemed sufficient to complete the scheduled sample size. The maximal duration for enrolment was thus set *a priori* at two years to prevent biases due to an exceedingly long recruitment period. Data was compared using the chi-square test, Fisher exact test, Student’s *t*-test and non-parametric Wilcox on test as appropriate (SPSS 18.0 for Windows, Chicago, IL, USA). A logistic regression model was used to adjust for age and baseline variables found to differ between the study groups. A binomial distribution model was used to calculate the 95%CI of proportions.

The perspective of the cost analysis was the one of the health provider, i.e. costs supported by the public health system. Costs supported by the individuals (transports, absences from work, etc.) were excluded. All considered costs were estimations. We included those for pharmacological compounds and those for the IVF procedure. The former were obtained through the consultancy of the website of the official Italian Institute for drugs AIFA (*Agenzia Italiana del Farmaco*) [8]. They relate to the price of the entire boxes (the costs of the non-used ampoules or tablets were not subtracted). Costs for the IVF cycles were derived from the regional Drugs Related Groups (DRG) costs [9]. They were as follows: 225 Euros for cycle preparation and monitoring; 2,232 Euros for oocytes collection; and 2,194 Euros for embryo-transfer. Only drugs administered domiciliary were included in the costs (Clomid®, Gonal-F®, Decapeptyl®, Ovitrelle®, Progeffik®). Those administered in the hospital were excluded since already part of the DRG costs of the IVF procedures. Costs of the ultrasound (US) scans and serum tests were excluded for the same reason. Costs related to pregnancy assistance were excluded from the model. The direct and indirect costs supported by the patients and their partners for referrals were also excluded.

## Results

**Figure 1:**
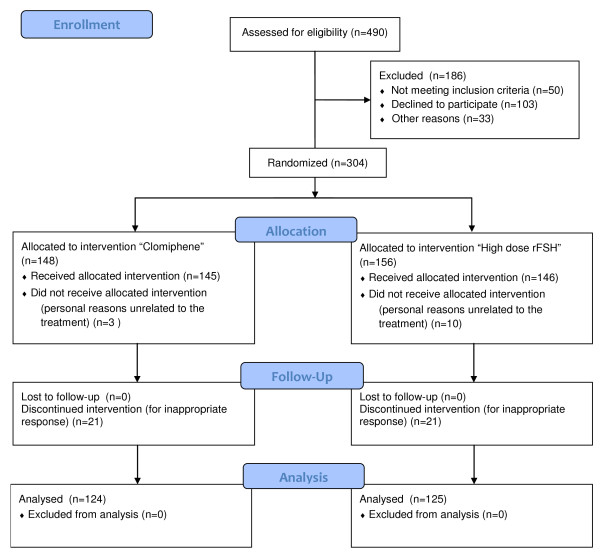
Flow chart of the study.

The study was interrupted after the scheduled two years of recruitment before reaching the sample size of 472 subjects. At this time, 304 women (64% of those scheduled) had been enrolled: 148 were allocated to clomiphene citrate and the remaining 156 to the short protocol with high doses of gonadotropins. Thirteen women (three in the clomiphene citrate group and ten in the high-dose gonadotropins group) dropped out prior to initiating the cycle for reasons unrelated to the prescribed regimen, leaving 291 women for data analysis. The study power was thus 60% rather than 80% as initially planned. The precise flow-chart of the study is shown in Figure 1.

Baseline characteristics of the women in the two study groups are shown in Table 1. No statistically significant differences emerged with the exception of the duration of infertility that was slightly longer in women allocated to high-dose gonadotropins. Twenty-one cycles per study group were cancelled during the stimulation (Table 2). Reasons for cancellation (Figure 1) were identical: no follicular growth (21 and 21 women), arrested follicular growth (3 and 3 women) and premature luteinisation (7 and 7 cases). All women in the clomiphene citrate group assumed the whole prescribed regimen. In women undergoing the short protocol with high doses of gonadotropins, the mean total dose of recombinant FSH given was 4029±1279 IU. Cycle outcome is illustrated in Table 2. A significantly higher number of follicles developed with the administration of high-dose gonadotropins (p<0.001) and a higher number of oocytes were retrieved during ovum pick-up (p<0.001). A lower proportion of women in the clomiphene citrate group performed the embryo transfer (p=0.008). The mean number of transferred embryos was similar between the group seven if it tended to be higher in the high-dose gonadotropins group (p=0.06). The clinical pregnancy rate per started cycle in the clomiphene citrate and high-dose gonadotropins groups was 5% (95%CI: 2-10%) and 6% (95%CI: 3-11%), respectively (p=1.00). The implantation rate was similar. One twin pregnancy was recorded in the clomiphene citrate group while one twin and one triplet pregnancy occurred in the high-dose gonadotropins group: no vanishing twins were observed and all multiple pregnancies ended in delivery of viable infants. The delivery rate did not differ. It was 3% (95%CI: 1-7%) and 5% (95%CI: 2-9%) in the clomiphene citrate and high-dose gonadotropins groups, respectively (p=0.77). The risk difference of delivery was +0.02 (95%CI: -0.03 to +0.07) for the high-dose gonadotropins group. The Odds Ratio (OR) of delivery for the clomiphene citrate group compared to high-dose gonadotropins group and adjusted for age and duration of infertility was 0.80 (95%CI: 0.25-2.63).

**Table 1:** Baseline characteristics of the study groups

**Characteristics**	**Clomiphene (n=145)**	**High dose rFSH (n=146)**	**p**
*Age (years)*	38.6 ± 2.9	38.5 ± 3.1	0.76
*BMI (Kg/m*^*2*^*)*	22.0 ± 2.7	22.0 ± 3.0	0.95
*Duration of infertility (years)*	3.5 ± 2.0	4.1 ± 2.2	0.02
*Smoking*	29 (20%)	27 (18%)	0.74
*Previous pregnancies*	42 (29%)	46 (32%)	0.69
*Previous ovarian surgery*	30 (21%)	27 (18%)	0.64
*Cycle length (days)*	27.2 ± 3.0	27.4 ± 2.4	0.71
*Previous IVF cycles*	44 (30%)	37 (25%)	0.34
*Day 3 serum FSH (IU/ml)*	14.6 ± 8.9	13.6 ± 9.0	0.68
*AMH (ng/ml)*	0.71 ± 1.0	0.76 ± 1.0	0.67
*Total AFC*	3.4 ± 2.5	3.9 ± 2.6	0.10

Data are expressed as mean ± SD or number (percentage).

**Table 2:** IVF-ICSI cycle outcome in the two study groups

**Characteristics**	**Clomiphene (n=145)**	**High dose rFSH (n=146)**	**p**
*Cancelled cycles*	21 (14%)	21 (14%)	1.00
*Number of follicles > 15 mm*^*a*^	1.7 ± 1.1	2.8 ± 1.9	<0.001
*Total number of follicles > 10 mm*^*a*^	2.5 ± 1.4	4.1 ± 2.6	<0.001
*Number of oocytes retrieved*^*a*^	1.1 ± 1.1	2.0 ± 1.8	<0.001
*0*	52 (42%)	29 (23%)	
*1-2*	58 (47%)	57 (46%)	<0.001
*≥ 3*	14 (11%)	39 (31%)	
*Fertilisation rate*^*b*^	75% (93/124)	70% (164/236)	0.33
*Women performing Embryo Transfer*	56 (39%)	79 (54%)	0.008
*Number of embryos transferred*^*c*^	1.5 ± 0.7	1.7 ± 0.7	0.06
*1*	33 (59%)	33 (42%)	
*2*	18 (32%)	35 (44%)	0.14
*3*	5 (9%)	11 (14%)	
*Number of pregnant women*	8	9	
*PR per started cycle*	5% (8/145)	6% (9/146)	1.00
*PR per retrieval*	6% (8/124)	7% (9/125)	1.00
*PR per embryo transfer*	14% (8/56)	11% (9/79)	0.81
*Embryos implanted (implantation rate)*	11% (9/84)	9% (12/136)	0.64
*Number of live birth deliveries*	5	7	
*DR per started cycle*	3% (5/145)	5% (7/146)	0.77
*DR per retrieval*	4% (5/124)	6% (7/125)	0.78
*DR per embryo transfer*	9% (5/56)	9% (7/79)	1.00

Data are expressed as mean ± SD or number (percentage) or median (interquartile range) or percentage (ratio).

^a^ Refers to women who underwent oocyte retrieval.

^b^ Refers to all oocytes used.

^c^ Refers to women performing embryo transfer.

PR: Clinical pregnancy rate. DR: Delivery Rate.

All the analyses were repeated considering separately women whose diagnosis of compromised ovarian reserve was based on a previous poor response cycle (≤ 3 oocytes with a conventional stimulation protocol) and those whose diagnosis was based exclusively on persistent elevated day 3 serum FSH. In these two subgroups, baseline characteristics according to treatment allocation did not differ (data not shown). The cycle outcome is summarised in Table 3. Overall, results from this analysis were in line with those observed for the entire cohort. Finally, we retrospectively evaluated the proportion of the recruited women who could be classified as poor responders before study entry according to the new definition proposed by the Consensus Conference of Bologna [4]. We found that at least 278 of them (96%) fulfilled these criteria. All the analyses were repeated focussing exclusively on this subgroup of women and results were similar (data not shown).

**Table 3:** IVF-ICSI cycle outcome in the two study groups

	**Previous Poor response**			**Day 3 serum FSH > 12 UI/ml**		
	**Clomiphene (n=120)**	**High dose rFSH (n=118)**	**p**	**Clomiphene (n=25)**	**High dose rFSH (n=28)**	**p**
*Cancelled cycle*	16 (3%)	18 (15%)	0.67	5 (20%)	3 (11%)	0.29
*Number of oocytes retrieved*^*a*^	1.4 ± 1.1	2.8 ± 2.4	0.001	2.2 ± 0.8	2.8 ± 1.2	0.23
*Fertilisation rate*^*b*^	88% (83/94)	69% (132/190)	0.001	50% (10/20)	67% (31/46)	0.27
*Women performing embryo transfer*	50 (42%)	61 (52%)	0.12	6 (24%)	18 (64%)	0.004
*Number of embryos transferred*^*c*^	1.5 ± 0.7	1.8 ± 0.7	0.08	1.3 ± 0.5	1.6 ± 0.6	0.33
*PR per started cycle*	7% (8/120)	7% (8/118)	0.97	0% (0/25)	4% (1/28)	0.53
*PR per embryo transfer*	16% (8/50)	13% (8/61)	0.44	0% (0/6)	6% (1/18)	0.75
*Embryos implanted (implantation rate)*	12% (9/76)	9% (10/107)	0.63	0% (0/8)	7% (2/29)	1.00
*DR per started cycle*	4% (5/120)	5% (6/118)	0.74	0% (0/25)	4% (1/28)	0.53
*DR per embryo transfer*	10% (5/50)	10% (6/61)	1.00	0% (0/6)	6% (1/18)	0.75

Data are expressed as mean ± SD or number (percentage) or percentage (ratio).

^a^ Refers to women who underwent oocyte retrieval.

^b^ Refers to all oocytes used.

^c^ Refers to women performing embryo transfer.

PR: Clinical pregnancy rate. DR: Delivery Rate.

During the study period, the delivery rates in the four participating units ranged from 16 to 20%, in line with the data of the national Italian Assisted Reproductive Technologies (ART) register [10]. All delivered infants were healthy. In order to identify predictive factors of delivery in our population, we performed a logistic regression analysis including baseline characteristics of the patients and study arm as covariates. None of them showed a significant association with delivery (data not shown). All the analyses were repeated separately for the four participating centres and results were similar (data not shown). No adverse events were observed during the trial in both groups of intervention.

The different invoice costs for the two therapeutic regimens are shown in Table 4. The mean costs per cycle were 2,803 and 5,423 Euros for the clomiphene citrate and high-dose gonadotropins groups, respectively. The mean per-cycle cost-saving resulting from using clomiphene citrate was thus 2,620 Euros, which would allow financing of an additional cycle with clomiphene citrate for every 1.03 cycles with the use of high-dose gonadotropins. The mean costs per delivery were 81,294 and 113,107 Euros in the clomiphene citrate and high-dose gonadotropins groups, respectively. The costs required to achieve an additional delivery in the high-dose gonadotropins group was 192,639 Euros. A sensitivity analysis based on the 95%CI of the DRs in the two groups was not conclusive. At one extreme, costs per delivery in the clomiphene citrate and high-dose gonadotropins groups were 41,000 and 271,100 Euros, respectively. At the opposite extreme, they were 60,300 for the latter group and 280,300 Euros for the former one (Table 4).

**Table 4:** Costs per cycle in the two study groups

**Items**	**Clomiphene (n=145)**	**High-dose rFSH (n=146)**
*Medications*		
*GnRH agonists*	-	13,434
*Recombinant-FSH*	-	320,604
*Clomiphene citrate*	1,459	-
*Progesterone*	655	660
*Subtotal for medications*	*2,113*	*334,698*
*Technical procedures*		
*Cycle preparation and monitoring*	32,625	32,850
*Oocyte retrieval*	248,868	250,875
*Embryo transfer*	122,864	173,326
*Subtotal for procedures*	*404,357*	*457,051*
*Total costs*	406,470	791,749
*Mean cost per patient*	2,803	5,423
*Mean cost per delivery (95%CI)*^*a*^	81,294 (40,046-280,324)	113,107 (60,254-271,146)

Costs are reported in Euros.

^a^ The 95%CIs were calculated based on the 95%CI of the delivery rate.

## Discussion

In women with compromised ovarian reserve selected for IVF, clomiphene citrate and a short protocol with high-dose gonadotropins are both associated with disappointing results. The use of the latter regimen increased the number of oocytes retrieved and the proportion of women performing embryo transfer but not the delivery rates. In fact, a regimen of ovarian stimulation using exclusively clomiphene citrate may be considered in the management of these women since the chances of success did not markedly differ and this scheme is associated with lower costs.

The disappointing chances of success in women with compromised ovarian response are important data emerging from our trial. In both arms of the study, the delivery rate was below 5%, corresponding to the definition of “very poor prognosis” recently proposed by the Ethics Committee of the American Society for Reproductive Medicine [11]. One may argue that this may be at least in part due to the low quality of the units involved. Even if we cannot robustly refute this criticism, some contrary arguments deserve to be mentioned. Firstly, during the study period, the participating centres reported delivery rates per started cycle for patients not included in the trial in line with those of the national Italian ART register. Secondly, all four units involved handled a consistent number of couples annually (>1000 IVF cycles per year) and all of them had a long-standing clinical and scientific experience in IVF. Thirdly, the observed low rate of success appears to be in agreement with data from the literature on women with a compromised ovarian reserve [12]. Finally, it should be emphasised that the criteria used for the definition of compromised ovarian reserve in this study were very stringent and we may have selected a subgroup of women whose ovarian reserve is particularly damaged. From the health-provider point of view, the appropriateness of this intervention is inevitably questionable in these cases. Of note, our economical model under-estimated the overall costs per delivery since it did not include the costs of pregnancy assistance. More in-depth economic analyses are required prior to drawing firm indications for guiding health provider’s decisions in these cases.

Some aspects of our trial warrant clarification and discussion. Of utmost relevance here is that the trial was closed prior to completing the scheduled sample size and the power of the study is 60% in contrast to the initial calculation. Once the sample size has not been achieved one cannot make definite conclusions. This is especially true when the there is no difference between the two arms indicating the possibility of a beta error. In fact, the upper limit of the absolute reduction difference in favour of the high-dose gonadotropins group (7%) exceeded the 5% limit postulated as clinically relevant. The incapacity to achieve the scheduled sample size occurred despite the number of eligible patients being adequate to fulfil recruitment within a two-year period (Figure 1) and despite the initial assumptions for sample size calculation not being stringent. In our view, the difficulty in recruitment reflects the negative prejudgements women may have on an innovative approach using exclusively clomiphene citrate. External conditioning from other infertile women, the media or the internet may have played a role on this. We also cannot rule out that at least some physicians engaged in recruitment were somewhat reluctant to our study design, thus unconsciously influencing the woman’s decision to decline participation. Future studies aimed at testing mild approaches for women with compromised ovarian reserve should consider in advance this difficulty. Results of the present study may be of help in this regard since they can be used to persuade physicians and eligible subjects about the appropriateness of alternative, less aggressive approaches. In this study, we planned a two-year period of recruitment in order to maintain the conditions of the studied population as stable as possible. This point is of particular relevance in the field of IVF since notions and practices are in rapid and constant evolution. However, given the difficulties that we encountered, in future trials, one may consider the possibility of extending the recruitment period beyond the conventional limit of two years despite the intrinsic risk of biases associated with prolonged enrolments. Unfortunately, difficulties in recruitment do not allow us to draw definite conclusions regarding the financial aspects. The sensitivity analysis using the limits of the 95%CI of the DRs was not conclusive because at the two extremes, there were relevant economic advantages of one of the two groups. As such, our data has to be intended as preliminary and further study is warranted to clarify whether clomiphene citrate should become the first-line option in women with compromised ovarian reserve.

Some further aspects of the trial deserve to be noted. Firstly, we decided to test clomiphene citrate rather then natural cycle IVF because this is currently the cheapest available option and because we had encouraging results in a preliminary study with this approach [5]. The underlying hypothesis tested is that, in a situation of compromised ovarian reserve, the regimen of stimulation is much less relevant than the patient herself in determining the success of IVF. Natural cycle IVF was a plausible alternative since it may appear at *prima facie* cheaper but monitoring these cycles is more troublesome because of the risk of premature LH surge and this may actually increase costs. In fact, some authors have suggested employing a natural-cycle-guided IVF adding GnRH antagonists and low-dose gonadotropins during the last days of stimulation to prevent this deleterious event [13]. However, this is inevitably more expensive. Overall, we deemed that clomiphene citrate represented the less expensive option. It is noteworthy that premature LH surge is less likely with this drug because of its long-lasting anti-oestrogen effects [14]. Accordingly, the rate of premature luteinisation was similar in the two arms of our study. The interest of clomiphene citrate in this context is further supported by the observation that the rate of cancellation was identical in the two arms of our trial. In fact, although discarded in clinical practice, IVF using exclusively clomiphene citrate can yield reasonable results. A clinical pregnancy rate per started cycle and per embryo transfer of 18% and 34% have been reported [15].

Secondly, the choice of a short protocol regimen as a comparator may also be a concern. As mentioned earlier, the most appropriate regimen for poor responders has yet to be established. Evidence on this point is confusing. Nevertheless, there is some data suggesting that the short protocol regimen may be more suitable in poor responders [3].

Thirdly, the inclusion criteria used to define poor responders may also be a point of debate. Until recently, a shared definition of this condition was lacking. However, in 2010, opinion leaders in this area met within an ESHRE consensus meeting in order to propose a unique definition [4]. The presence of at least two of the following features are now necessary to define poor responders: 1) advanced maternal age (≥40 years) or any other risk factors for poor response;2) previous poor ovarian response (≤3 oocytes retrieved with a conventional stimulation protocol);and 3) an abnormal ovarian reserve test (AFC<5-7 follicles or AMH<0.5-1.1 ng/ml). Two episodes of poor response after maximal stimulation is an additional condition to define poor responders. In our study, we could not use these criteria since they were published after the study was implemented. However, it has to be emphasised that at least 278 women (96%) fulfilled this definition. An additional questionable point in this regard is the use of serum FSH as a unique selection criterion for a subgroup of included subjects. More than one criterion to characterise a woman as a poor responder is generally recommended [16]. However, it has to be pointed out that the use of serum FSH to predict poor responsiveness is arguable because of a high rate of false negatives (normal FSH but poor response) rather than the opposite (high FSH but normal response). Serum FSH is actually a late marker of compromised ovarian reserve. In fact, our decision led us to exclude some women with compromised ovarian reserve rather than including subjects with normal ovarian reserve. The analysis focussing exclusively on women recruited based on elevated serum FSH (see Table 3) confirmed this view. On the other hand, the selection criteria has to be clearly kept in mind in the interpretation of our results and it has to be remembered that inferences should be done exclusively to the specific population in our study.

Fourthly, the study was un-blinded. However, the impact of this potential bias is presumably small because the funding source was impartial to the treatments studied and a relevant role of the placebo effect in this kind of context is questionable [17]. Finally, it is worth mentioning that baseline characteristics of the two groups were similar, thus supporting the view that randomisation effectively prevented biases in allocation in our trial. Only duration of infertility slightly differed. However, this variable was not found to influence the chances of success by logistics regression analysis, thus excluding a critical confounding effect.

## Conclusions

In conclusion, we failed to document marked differences between clomiphene citrate and a short protocol with high doses of gonadotropins in women requiring IVF who were diagnosed with a compromised ovarian reserve based on day 3 serum FSH persistently above 12 IU/ml or a history of previous poor response (≤ 3 oocytes with a conventional stimulation protocol). Given the lower costs associated with the use of clomiphene citrate, this regimen may be considered in the management of these women. However, further evidence is required mainly because of the unusually low delivery rates observed in our trial. In fact, this exceedingly low rate of success and the relatively small sample size does not permit a valid statistical non-inferiority assessment. Moreover, economic analyses cannot be viewed as definite because of the particular and unusual criteria used to select our population. External validation of the low success rate in this particular group of women is thus necessary prior to confirm the unfavourable profile of our costs calculations.

## Competing interests

The authors declare that they have no competing interests.

## Authors' contributions

RG Study design, critical discussion. LSPE Study design, critical discussion. FR Study design, critical discussion. BC Study design, execution, critical discussion. SC Execution, critical discussion. AF Recruitment, execution. AV Recruitment , execution. MRM Recruitment, execution. CM Study design, critical discussion. PA Execution, analysis, manuscript drafting. SE Study design, analysis, manuscript drafting and critical discussion. All authors read and approved the final manuscript.
